# Trends of drug shortages in China from 2018 to 2020: A retrospective observational study

**DOI:** 10.7189/jogh.14.04225

**Published:** 2024-10-11

**Authors:** Tao Huang, Lin Bai, Huangqianyu Li, Hao Li, Zhiyuan Wang, Luwen Shi, Xiaodong Guan

**Affiliations:** 1 Department of Pharmacy Administration and Clinical Pharmacy, School of Pharmaceutical Sciences, Peking University, Beijing, China; 2 International Research Center for Medicinal Administration, Peking University, Beijing, China

## Abstract

**Background:**

Drug shortage is a significant global public health challenge, threatening both drug safety and patient health. While several studies concerning this issue have been conducted in the USA and in European countries, research in China remains limited. In this study, we aimed to analyse the characteristics and trends of drug shortage in China.

**Methods:**

We obtained monthly data reporting drug shortages from January 2018 to December 2020 from 1124 medical institutions in 258 prefecture-level cities. We described the characteristics of drugs reported to be in shortage and calculated the occurrence, geographical coverage, and duration of drug shortages. We conducted the modified Mann-Kendall test to analyse trends in drug shortages at the city level over time.

**Results:**

We identified 2368 drugs from 121 814 shortage records. During the study period, the mean monthly occurrence of shortages for each drug was 1.2 (standard deviation (SD) = 2.5), affecting on average 2.8 (SD = 4.0) cities, with a mean duration of 3.6 (SD = 5.7) months. Around half of the shortages influenced only one city (n = 19 215, 51.6%) and lasted only one month (n = 19 024, 55.8%). The occurrence (z-statistic (Z) = −3.83; *P* < 0.001), geographical coverage (Z = −2.02; *P* = 0.043), and duration (Z = −3.10; *P* = 0.002) of shortages showed decreasing trends over time. However, there was no significant change in the occurrence (Z = 0.90; *P* = 0.367), geographical coverage (Z = 1.70; *P* = 0.090), or duration (Z = −1.64; *P* = 0.102) of emergency medication shortages during the study period.

**Conclusions:**

Decreasing trends were observed for the occurrence, geographical coverage, and duration of drug shortages in China; most were observed only in limited geographical areas and lasted short periods. More efforts are needed to alleviate the shortage of emergency and antineoplastic medicines.

Drug shortages are a major public health threat worldwide [1–3], frequently leading to suboptimal treatment, increased medical costs, and increased risks of death for patients, particularly in developing countries with limited medical sources [4–7]. It was estimated that 38% of medical errors and an additional USD 359 million per year were directly related to drug shortages [8]. Despite measures to alleviate this issue [9], such as the 2012 Food and Drug Administration Safety and Innovation Act in the USA [10], shortages remained and even increased, especially for injections, gastroenterology medicines, and emergency medicines [11–14]. The most recent studies in European countries and the USA showed that over 95% of pharmacists have witnessed or are witnessing drug shortages [12,15].

China, one of the largest and most populous developing countries, also suffers drug shortages, such as those of cytarabine and trastuzumab in 2018 [16–18]. In contrast to the USA and other developed countries, where numerous studies have investigated the full scope and changing trends of drug shortages [11–15,19], limited evidence is available for China [2]. Moreover, drug shortages in developing countries can have more serious clinical implications compared to developed countries, due to a lack of approved alternative treatments and limited capacity to predict and detect drug shortages [20]. For this reason, like many developed countries, China established a national drug shortage reporting system and a strategic national stockpile programme, as well as released a national catalogue of critical drugs on shortage [2,21–24]. Estimating the magnitude and identifying the changing trends of drug shortages in China may reveal the effectiveness of these policy tools, providing references for the design of relevant policies in developing countries.

In this study, we retrospectively described the characteristics of and trends in reported drug shortages in China using national records from 1124 hospitals in 256 prefecture-level cities between 2018 and 2020.

## METHODS

### Data source

The China Medical Economic Information (CMEI) database of the Chinese Pharmaceutical Association has been collecting monthly data on drug shortages from hospitals across the country. In this context, a shortage is defined as a drug either being out of stock or not being supplied on time at the reporting hospital during the month. A total of 1124 hospitals that consecutively reported drugs in shortage every month between 2018 and 2020 were sampled. These hospitals covered 258 cities across 31 provinces in China, including four municipalities (Beijing, Tianjin, Shanghai and Chongqing) and 254 prefecture-level cities (spanning 76.3% of the total 333 prefecture-level cities in China) [25]. The CMEI was responsible for checking the completeness of the reported data monthly.

#### Data collection and processing

We obtained monthly drug shortage data between 2018 and 2020 from the CMEI database. This information included the hospital name, city name, and shortage month, as well as information on the drug reporting shortage, including the generic name of the drug, dosage form, and route of administration. Dosage forms were grouped into aerosol, capsule, drop, emulsion, gel, granule, liquid, ointment, patch, pill, powder, solution, suppository, suspension, syrup, tablet, tincture, and miscellaneous forms. Routes of administration were categorised as buccal, cutaneous, haemodialysis, inhalation, nasal, ophthalmic, oral, optic, parenteral, rectal, sublingual, and vaginal. We identified therapeutic areas of each drug according to the World Health Organization Anatomical Therapeutic Chemical Classification [26] and classified whether the drug was designated as an emergency medicine based on the list published by the Chinese Medical Association and the Chinese Medical Doctor Association [27].

We considered products with the same generic name, dosage form, and route of administration as the same drug. We then aggregated hospital-reported shortage data to create city-level summaries based on the location of the hospitals for further analysis. If a hospital reported a drug shortage, that drug was considered to be in shortage for the entire city during the report month (Figure S1 in the **Online Supplementary Document**).

### Outcome measures

Three outcomes were calculated using the city-level data to capture the landscape of drug shortages in China (Figure S1 in the **Online Supplementary Document**). First, we calculated the occurrence of reported shortages of drugs by calculating the number of times that they were reported to be in shortage by cities during the month. Second, we assessed the geographical coverage of the shortage of every drug each month by calculating the number of cities reporting that the drug was in shortage during the month. Third, we assessed the duration of each drug shortage in each city by counting the number of consecutive months when the drug was reported to be in shortage in that specific city.

### Statistical analysis

We used descriptive analyses to describe the characteristics of drugs that experienced shortages during the study period, presenting continuous variables as means (x̄) and standard deviations (SDs) and categorical variables as numbers and proportions. Outcomes, including the overall and monthly occurrences, average geographical coverages, and average durations of drug shortages, were calculated for all drugs. We conducted the modified Mann-Kendall test to analyse the changing trend in the monthly occurrence, average geographical coverage, and average duration of drug shortages during the study period [28]. To avoid bias stemming from the possibility that shortages starting earlier would expect longer duration, we performed trend tests for the duration of shortages after excluding data from the first and last six months (almost one SD of average shortage duration) of the study period. Subgroup analyses were conducted based on the dosage form of the drug, route of administration, therapeutic area, and whether it was included on the emergency list.

We performed all analyses in Microsoft Excel 2019 (Redmond, Washington, USA) and R, version 4.3.1 (R Core Team, Vienna, Austria). A two-sided *P* < 0.05 was considered statistically significant.

## RESULTS

### Characteristics of drugs in shortage

Overall, 121 814 drug shortage records and 2368 drugs reporting shortages were identified in 258 cities between 2018 and 2020 (**Table 1**). Among all drug shortage records, the most frequently observed dosage forms were solutions (n = 42 203, 34.6%), tablets (n = 42 150, 27.7%), and powders (n = 22 689, 18.6%). The most frequently observed routes of administration were parenteral (n = 59 004, 48.4%) and oral (n = 49 539, 40.7%). Among all therapeutic areas, drugs for antineoplastic and immunomodulating agents (n = 14 587, 12.0%) and anti-infectives for systemic use (n = 12 160, 10.0%) contributed to a little over a fifth of all shortage records. Emergency medicine drugs comprised 11.1% (n = 13 564) of all drug shortage records. Drugs that were most frequently reported to be in shortage were methotrexate, allopurinol, ketamine, cytarabine, nitroglycerin, vincristine, atropine, vitamin K1, and posterior pituitary and pyridostigmine bromide (Table S1 in the **Online Supplementary Document**). Notably, seven out of these ten drugs were administered via parenteral injections.

**Table 1:** Characteristics of reported shortages and included drugs

Characteristics	Reported shortage (n = 121 814), n (%)	Drug (n = 2368), n (%)
Dosage form		
*Solutions*	42 203 (34.6)	655 (27.7)
*Tablets*	42 150 (34.6)	702 (29.6)
*Powders*	22 689 (18.6)	391 (16.5)
*Capsules*	5858 (4.8)	272 (11.5)
*Ointments*	2312 (1.9)	51 (2.3)
Others*	6602 (5.4)	297 (12.5)
Route of administration		
*Parenteral*	59 004 (48.4)	829 (35.0)
*Oral*	49 539 (40.7)	1142 (48.2)
*Cutaneous*	4525 (3.7)	175 (7.4)
*Ophthalmic*	4019 (3.3)	101 (4.3)
*Vaginal*	1607 (1.3)	45 (1.9)
*Others*†	3120 (2.6)	76 (3.2)
Therapeutic area		
*Alimentary tract and metabolism*	16 904 (13.9)	397 (16.8)
*Nervous system*	16 502 (13.5)	289 (12.2)
*Cardiovascular system*	15 633 (12.8)	235 (9.9)
*Antineoplastic and immunomodulating agents*	14 587 (12.0)	209 (8.8)
*Anti-infectives for systemic use*	12 160 (10.0)	284 (12.0)
*Others*‡	46 028 (37.8)	954 (40.3)
Emergency medicine	13 564 (11.1)	83 (3.5)

*Aerosol, drop, emulsion, gel, granule, liquid, patch, pill, suppository, suspension, syrup, tincture, and miscellaneous forms.

†Buccal, haemodialysis, inhalation, nasal, optic, rectal, and sublingual.

‡Antiparasitic products, insecticides and repellents, blood and blood-forming organs, dermatological, genitourinary system and sex hormones, musculoskeletal system, respiratory system, sensory organs, systemic hormonal preparations (excluding sex hormones and insulins), and others.

### Monthly number of reported drug shortages

**Figure 1:**
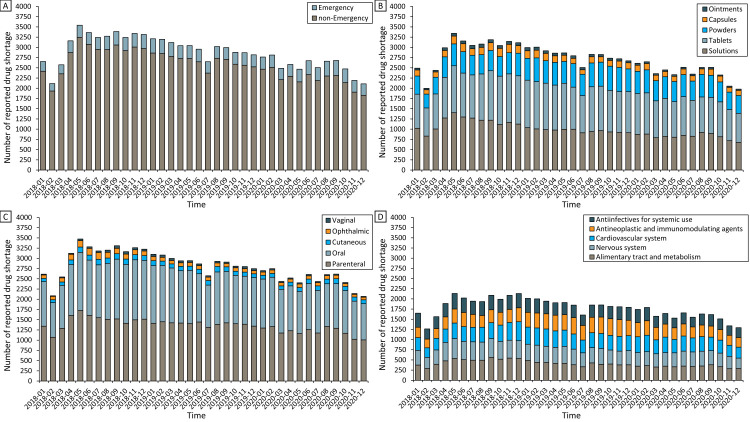
Trends in the monthly number of reported shortages for all drugs and different subgroups between 2018 and 2020: **Panel A**. Emergency medicines and non-emergency medicines. **Panel B.** Dosage forms. **Panel C.** Route of administrations. **Panel D.** Therapeutic areas.

The monthly occurrence of all drug shortages in the 258 cities showed a decreasing trend of 20.6%, from 2655 times in January 2018 to 2109 times in December 2020 (z-statistic (Z) = -3.83; *P* < 0.001) (**Figure 1**, panel A; Table S2 in the **Online Supplementary Document**). Decreasing trends in the monthly numbers of reported shortages were also observed in the subgroup analyses of the five most commonly reported dosage forms, routes of administration, and therapeutic areas, except for drugs that are in the powder form (Z = 0.81; *P* = 0.416), administered via cutaneous (Z = -1.48; *P* = 0.139) and vaginal routes (Z = -1.54; *P* = 0.123), and for the therapeutic areas of the cardiovascular system (Z = -0.74; *P* = 0.485) and antineoplastic and immunomodulating agents (Z = -0.13; *P* = 0.900). We further observed a non-significant increasing trend for the monthly number of shortages of emergency medicine (Z = 0.90, *P* = 0.367).

### Geographical coverage of reported shortages

**Figure 2:**
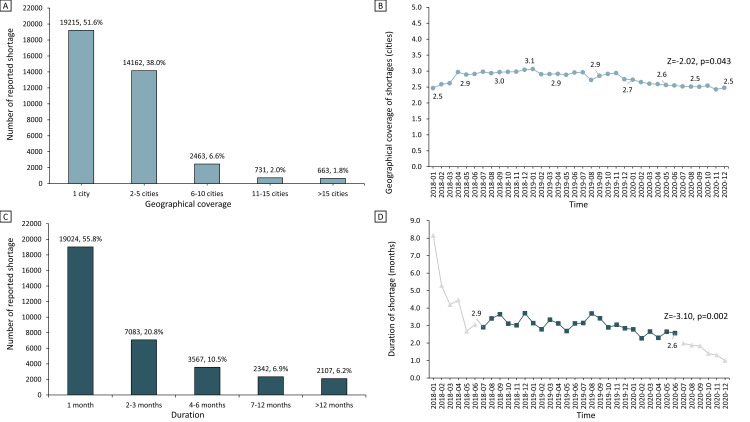
Distribution of and trends in geographical coverage and duration of reported shortages from 2018 to 2020: **Panel A**. Number of reported shortages for different levels of geographical coverage. **Panel B.** Monthly trends in the average geographical coverage of reported shortages. **Panel C.** Number of reported shortages for different durations. **Panel D.** Monthly trends in the average duration of reported shortages. To avoid possible bias stemming from the possibility that shortages starting earlier would expect longer duration, trend tests for the duration of shortages were performed after excluding data from the first and last six months (almost one standard deviation of average shortage duration) of the study period.

Each month, the drug shortages affected between 1 and 67 cities across 258 cities in China, with a mean of 2.8 (SD = 4.0) cities experiencing shortages. Among all drug shortages, around half (n = 19 215, 51.6%) of shortages influenced only one city, while 10.4% (n = 3857) of shortages covered more than five cities (**Figure 2****,** panel A).

**Figure 3:**
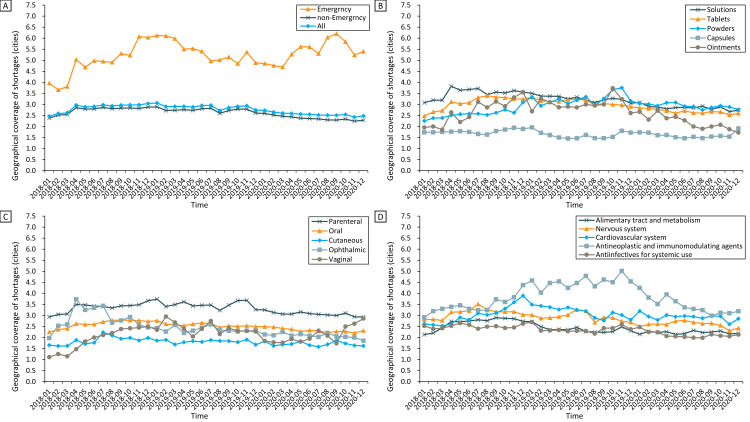
Monthly trends in the geographical coverage of reported shortages for different subgroups from 2018 to 2020: **Panel A**. Emergency medicines and non-emergency medicines. **Panel B.** Dosage forms. **Panel C.** Route of administrations. **Panel D.** Therapeutic areas.

The overall average shortage coverage in the 258 cities showed a downward trend from 2018 to 2020 (Z = −2.02; *P* = 0.043) (**Figure 2**, panel B; Table S2 in the **Online Supplementary Document**). Similar decreasing trends were observed in the subgroup analyses of the five most commonly reported dosage forms, routes of administration, and therapeutic areas during the study period, except for drugs that were in the powder forms (Z = 1.30; *P* = 0.192), administered via parenteral (Z = -1.16; *P* = 0.622) and vaginal routes (Z = −0.01; *P* = 0.995), and for the therapeutic areas of the cardiovascular system (Z = −0.74; *P* = 0.485) and antineoplastic and immunomodulating agents (Z = −0.13; *P* = 0.900) (**Figure 3**; Table S2 in the **Online Supplementary Document**). A statistically insignificant increase in the coverage of shortages of emergency medicine was observed (Z = 1.70; *P* = 0.090) (**Figure 3****,** panel A).

### Duration of reported shortages

Overall, the duration of drug shortages in the 258 cities was between 1 and 36 months, with a mean of 3.6 months (SD = 5.7). Among drug shortages, around half had a duration of one month (n = 19 024, 55.8%), while 13.1% (n = 4449) lasted more than six months (**Figure 2****,** panel C).

**Figure 4:**
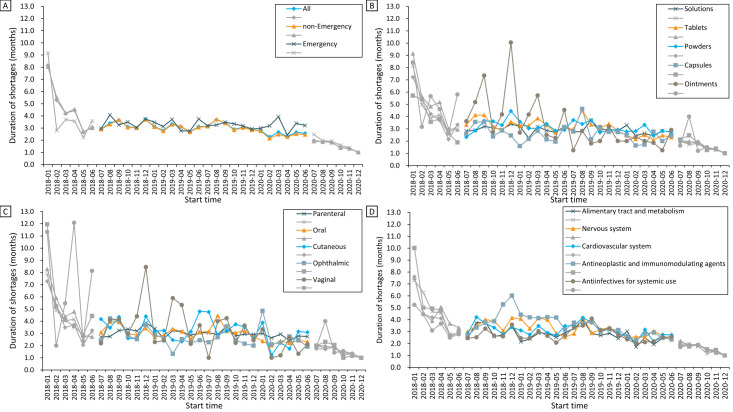
Monthly trends in the duration of reported shortages for different subgroups from 2018 to 2020: **Panel A**. Emergency medicines and non-emergency medicines. **Panel B.** Dosage forms. **Panel C.** Route of administrations. **Panel D.** Therapeutic areas. To avoid possible bias stemming from the possibility that shortages starting earlier would expect longer duration, trend tests for the duration of shortages were performed after excluding data from the first and last six months (almost one standard deviation of average shortage duration) of the study period.

Overall, the average drug shortage duration in the 258 cities showed a downward trend, with a decrease of 11.5% from 2.9 months in July 2018 to 2.6 months in June 2020 (Z = −3.10; *P* = 0.002) (**Figure 2****,** panel D; Table S2 in the **Online Supplementary Document**). Decreasing trends in the durations of reported shortages were also observed in the subgroup analyses, except for drugs that were in the forms of solutions (Z = −1.46; *P* = 0.143) and capsules (Z = −1.36; *P* = 0.172), administered via the cutaneous route (Z = −1.61, *P* = 0.107), and for the therapeutic areas of alimentary tract and metabolism (Z = -1.46; *P* = 0.143) and anti-infectives (Z = −0.51, *P* = 0.613) (**Figure 4**; Table S2 in the **Online Supplementary Document**). There was no significant decrease in the shortage duration of emergency medicine (Z = −1.64, *P* = 0.102) (**Figure 4****,** panel A).

## DISCUSSION

To the best of our knowledge, this is the first study to evaluate drug shortages across China using a nationally representative data of records reporting drug shortages. Our findings showed that the overall occurrence, geographical coverage, and duration of drug shortages showed decreasing trends between 2018 and 2020. After aggregating hospital-level into city-level data, the influence of over half of the drug shortages did not extend to more than one city or one month. However, drug shortages for emergency drugs did not show significant alleviation in either occurrence or coverage over time.

The overall geographical coverage of drug shortages in China was limited, and the average duration of 3.6 months was significantly shorter than that of six to nine months that have been reported by studies in the USA and Belgium [29–32]. This discrepancy may be attributed to the different causes of drug shortages in China, the USA, and European countries. For example, over 70% of drug shortages in the USA and Europe were caused by problems with drug manufacturing and lack of raw materials [33–38], while most drug shortages in China were caused by problems with drug tendering and pricing [35]. The limited geographical coverage of drug shortages in this study also indicated that temporary disruptions in the tendering, procurement, or distribution contributed to most drug shortages in China, as disruptions in pharmaceutical manufacturing usually resulted in national drug shortages, such as the national shortage of cytarabine and trastuzumab in 2018 [16–18]. Therefore, improving clarity and transparency of information about drug pricing, tendering, and inventory to better distribute drugs can help hospitals and cities recover from drug shortages in China [39,40].

Our results further highlight that drug shortages in China were alleviated despite disruptions rising from the coronavirus disease 2019 (COVID-19) pandemic, as shown by significant downward trends in the overall occurrence, geographical reach, and duration of drug shortages. This contradicts results from the USA and European countries, such as France and Italy [12,41,42]. According to the 2022 Food and Drug Administration Drug Shortage Report, the number of ongoing drug shortages nearly doubled from 2017 to 2020 [12]. A study based on the French national drug shortage reporting system also showed a 4-fold increase in drug shortages during the study period [41]. The difference in changing trends can be explained by the different reasons behind the drug shortages. Drug shortages in developed countries are mostly caused by a lack of raw materials and issues concerned with the quality of drug manufacturing, like the 2019 cefazolin shortage in Japan and the 2023 cisplatin shortage in the USA [43,44]. In contrast, drug shortages in China are usually caused by problems with drug tendering and pricing, and serval policy tools have proven effective in addressing these problems. To ensure a stable supply of drugs with small market demand and low prices, such as methimazole and digoxin, the Chinese government has been establishing designated factories to produce these drugs through bidding since 2012, which proved to increase drug availability [45]. Provinces also have their stockpile plans for drugs susceptible to shortages, including emergency medicines and drugs with small market demand, to further mitigate the effects of these shortages [9,46]. Additionally, the national drug shortage monitoring system and collaborative initiatives involving multiple stakeholders (including provincial regulators, drug distributors, and manufacturers) contributed to the earlier detection of and response to drug shortages, especially shortages that were derived from disruptions in the process of tendering, procurement, and distribution of drugs [46]. However, efforts are still needed to improve the transparency of designating drug factories and stockpile plans and to align these decisions with evidence in China. A previous study also showed complaints that collaborative efforts were time-consuming and often delayed response to short shortages, aside from a low sensitivity of detection of these shortages in the first place [47]. Therefore, systematic evaluations of the effectiveness of these policies and evidence-based efforts are urgently needed.

It is worth noting that the monthly occurrence and coverage of shortages of emergency medicine were not alleviated during the study period; instead, they showed upward trends, similar to findings observed in the USA [11,30]. Additionally, the geographical coverage of shortages of emergency medicines was wider than that of other drugs. Possible reasons may be that emergency medicines are generally lower priced and lack alternatives [48]. The unpredictable clinical demand for emergency medicines may worsen their shortage and make them harder to prevent and prepare for. Additionally, during the COVID-19 pandemic, the clinical demand for essential life-saving drugs (e.g. norepinephrine) increased while the manufacturing of these medicines was shut down due to quarantine measures, resulting in a serious imbalance between supply and demand [49]. Alongside current policies and interventions, actions like appropriate ration plans shall be taken to manage the emergency medicine shortage and to prepare for possible future shortage incidences [50].

We also discovered that nearly 10% of shortages influenced more than five cities and over 6% of shortages lasted for over a year. Qualitative research suggested that manufacturing problems and single-source providers are contributors to drug shortages with wider or more long-lasting influence [35,51], such as the shortage of methotrexate in 2019 that was derived from the discontinuation of one manufacturer. Our findings also suggested that the geographical coverage of shortages of drugs administered parenterally contributed the most to lasting shortages, consistent with results from other countries [19,52]. Seven out of ten drugs most frequently reported as being in shortage were administered via parenteral injections. Parenteral drugs are more difficult to produce and transport than other drugs and thus are more susceptible to disruptions in manufacturing and delivery [53]. Consistent with previous studies in the USA and Europe [41,52], anti-infective, cardiovascular, and antineoplastic drugs contributed significantly to the occurrence of drug shortages. The shortages of drugs like benzylpenicillin and vincristine are likely caused by a lack of market incentives and low profitability [54,55]. With increasing occurrence and geographical coverage of shortages of these drugs, they became a greater threat to public health [56,57]. Future research should seek to design targeted measures to reduce the risks these shortages pose to patient health.

### Strengths and limitations

The strength of our study is that, to our knowledge, it is the first estimate the nationwide magnitude and changing trends of drug shortages in China using data reporting drug shortages across the country. Previous studies were mostly conducted based on data from a single hospital or province obtained through questionnaires [35,48,58–60], making it hard to generalise the results to the entire country.

This study also has several limitations. First, caution should be taken while extrapolating our results to hospitals not included in our sample. Nevertheless, the sampled hospitals were from nearly 80% of all prefecture-level cities in China, spanning cities with different levels of economic development and health care resources, and thus our data set was nationally representative and provided the most comprehensive pool of drug shortage data during the study period. Second, the uncertainty of study results may arise from varying criteria for delayed supply. Sampled hospitals had differing definitions for timely drug restocking, preventing more accurate estimations of drug shortages in China. Still, the CMEI database is the largest in China, reporting drug shortages and providing the most detailed information. We also aggregated the data at the city level to further mitigate the effect of the varying criteria and enhance the robustness of our results. Third, our study design was not powered to draw causal effects between concerned policy tools and the number of drug shortages in China. Further studies using rigorous study designs, such as difference-in-difference or interrupted time-series analysis, are needed to evaluate the effect of these tools in different settings. Lastly, we lacked data on the characteristics of manufacturing, prices, and distribution of drug products. Future work needs to incorporate these factors to analyse the causes of drug shortages, especially for emergency and antineoplastic medicines.

## CONCLUSIONS

We found decreasing trends in the occurrence, geographical coverage, and duration of drug shortages between 2018 and 2020 in China. Most drug shortages observed covered limited geographical areas and lasted short periods. However, for emergency medicines with no therapeutic alternative available, continued efforts and interventions by the industry and the government are needed, such as mandating the reporting of production discontinuation and strategic stockpiling. Further studies should focus on understanding the root causes and estimating the social and patient losses due to drug shortages.

## Additional material


Online Supplementary Document

## References

[1] Fox ER Sweet BV Jensen V Drug shortages: a complex health care crisis. Mayo Clin Proc. 2014;89:361–73. 10.1016/j.mayocp.2013.11.014 24582195

[2] Acosta A Vanegas EP Rovira J Godman B Bochenek T Medicine Shortages: Gaps Between Countries and Global Perspectives. Front Pharmacol. 2019;10:763. 10.3389/fphar.2019.00763 31379565 PMC6658884

[3] Biedermann F Seeking solutions to global drugs shortages. Lancet. 2023;401:720–1. 10.1016/S0140-6736(23)00437-3 36871561

[4] Butterfield L Cash J Pham K Drug shortages and implications for pediatric patients. J Pediatr Pharmacol Ther. 2015;20:149–52. 10.5863/1551-6776-20.2.149 25964733 PMC4418683

[5] Bible JR Evans DC Payne B Mostafavifar L Impact of drug shortages on patients receiving parenteral nutrition after laparotomy. JPEN J Parenter Enteral Nutr. 2014;38:65S–71S. 10.1177/0148607114550317 25239113

[6] Hayes MS Ward MA Slabaugh SL Xu Y Lessons from the leucovorin shortages between 2009 and 2012 in a Medicare advantage population: where do we go from here? Am Health Drug Benefits. 2014;7:264–70. 25237422 PMC4163778

[7] Hughes KM Goswami ES Morris JL Impact of a Drug Shortage on Medication Errors and Clinical Outcomes in the Pediatric Intensive Care Unit. J Pediatr Pharmacol Ther. 2015;20:453–61. 10.5863/1551-6776-20.6.453 26766934 PMC4708954

[8] Vizient. Drug shortages and labor costs: measuring the hidden costs of drug shortages on U.S. hospitals. 2019. Available: https://wieck-vizient-production.s3.us-west-1.amazonaws.com/page-Brum/attachment/c9dba646f40b9b5def8032480ea51e1e85194129. Accessed: 23 February 2024.

[9] Vogler S Fischer S How to address medicines shortages: Findings from a cross-sectional study of 24 countries. Health Policy. 2020;124:1287–96. 10.1016/j.healthpol.2020.09.001 33032846 PMC7505130

[10] Food and Drug Administration. Food and Drug Administration Safety and Innovation Act (FDASIA). 2018. Available: https://www.fda.gov/regulatory-information/selected-amendments-fdc-act/food-and-drug-administration-safety-and-innovation-act-fdasia. Accessed: 23 February 2024.

[11] Chen SI Fox ER Hall MK Ross JS Bucholz EM Krumholz HM Despite Federal Legislation, Shortages Of Drugs Used In Acute Care Settings Remain Persistent And Prolonged. Health Aff (Millwood). 2016;35:798–804. Accessed February 23, 2024. 10.1377/hlthaff.2015.1157 27140985 PMC6712565

[12] Food and Drug Administration. Tenth Annual Report on Drug Shortages for Calendar Year 2022. Silver Spring, Maryland, USA: Food and Drug Administration; 2022. Available: https://www.fda.gov/media/169302/download. Accessed: 23 February 2024.

[13] Patel J Fox ER Zocchi M Lee ZE Mazer-Amirshahi M Trends in United States drug shortages for medications used in gastroenterology (2001-2014). J Med Access. 2017;1:e58–64.

[14] Mazer-Amirshahi M Fox ER Zocchi MS Pines JM van den Anker JN Longitudinal trends in U.S. shortages of sterile solutions, 2001-17. Am J Health Syst Pharm. 2018;75:1903–8. 10.2146/ajhp180203 30463866

[15] European Association of Hospital Pharmacists. EAHP 2023 Shortage Survey Report. Brussels, Belgium: European Association of Hospital Pharmacists; 2023. Available: https://www.acadpharm.org/dos_public/SHORTAGES_SURVEY_REPORT_FINAL.PDF. Accessed: 23 February 2024.

[16] National Health Commission. [Notice on ensuring the supply of cytarabine injection]. 2019. Available: http://yp.ynyyzb.com.cn/detail.html?infoId=10915&CatalogId=3. Accessed: 23 February 2024. Chinese.

[17] Network of Liaoning Province. [Notice on ensuring the supply of cytarabine injection of Liaoning Province: Medicine and Medical Consumables Centralized Procurement 2019]. Available: https://ypzb2015.lnypcg.com.cn/UploadFile/20190121_92951_6119.pdf. Accessed: 23 February 2024. Chinese.

[18] Beijing Youth Daily. Breast cancer 'life-saving drug' Herceptin is out of stock. 2018. Available: https://www.thepaper.cn/newsDetail_forward_2163884. Accessed: 23 February 2024.

[19] Food and Drug Administration. Drug Shortages CY 2022: U.S. Food and Drug Administration. Silver Spring, Maryland, USA: Food and Drug Administration; 2023. Available: https://www.fda.gov/media/169302/download. Accessed: 23 February 2024.

[20] Shukar S Zahoor F Hayat K Saeed A Gillani AH Omer S Drug Shortage: Causes, Impact, and Mitigation Strategies. Front Pharmacol. 2021;12:693426. 10.3389/fphar.2021.693426 34305603 PMC8299364

[21] Shi Y Yang P Li X Lv J Yin T Gong Z Combating drug shortages in China: surveillance warning and practice standardization. Int J Clin Pharm. 2020;42:309–14. 10.1007/s11096-020-00987-5 32048122

[22] National Health Commission. [Notice on issuing the National List of drugs on shortage National Health Commission]. 2020. Available: http://www.nhc.gov.cn/yaozs/s7653/202012/f30aad8ec4ba48a9afa2e559f4d20e7c.shtml. Accessed: 23 February 2024. Chinese.

[23] European Medicines Agency. First version of the Union list of critical medicines agreed to help avoid potential shortages in the EU. 2023. Available: https://www.ema.europa.eu/en/news/first-version-union-list-critical-medicines-agreed-help-avoid-potential-shortages-eu. Accessed: 23 February 2024.

[24] Health Affairs. Strategic Reserve Stockpiling: A Proposal To Prevent Critical Drug Shortages 2012. Available: https://www.healthaffairs.org/content/forefront/strategic-reserve-stockpiling-proposal-prevent-critical-drug-shortages. Accessed: 23 February 2024.

[25] Ministry of Civil Affairs of the People’s Republic of China. [Statistical table of administrative divisions of the People's Republic of China]. 2020. Available: http://xzqh.mca.gov.cn/statistics/2020.html. Accessed: 23 February 2024. Chinese.

[26] Norwegian Institute of Public Health. ATC/DDD Index 2024. 2024. Available: https://www.whocc.no/atc_ddd_index/. Accessed: 14 January 2024.

[27] National Health Commission. [Notice on the publication of selection principles and drug list (chemicals and biological products) for women and pediatric medicine and emergency medicine]. 2015. Available: http://www.nhc.gov.cn/yaozs/s7652/201509/105a04136fad412e9f57fa8034126bd9.shtml. Accessed: 23 February 2024. Chinese.

[28] Yue S Wang C The Mann-Kendall Test Modified by Effective Sample Size to Detect Trend in Serially Correlated Hydrological Series. Water Resources Management. 2004;18:201–18. 10.1023/B:WARM.0000043140.61082.60

[29] Quadri F Mazer-Amirshahi M Fox ER Hawley KL Pines JM Zocchi MS Antibacterial drug shortages from 2001 to 2013: implications for clinical practice. Clin Infect Dis. 2015;60:1737–42. 10.1093/cid/civ201 25908680

[30] Hawley KL Mazer-Amirshahi M Zocchi MS Fox ER Pines JM Longitudinal Trends in U.S. Drug Shortages for Medications Used in Emergency Departments (2001-2014). Acad Emerg Med. 2016;23:63–9. 10.1111/acem.12838 26715487

[31] Bauters T Claus BOM Norga K Huys I Simoens S Laureys G Chemotherapy drug shortages in paediatric oncology: A 14-year single-centre experience in Belgium. J Oncol Pharm Pract. 2016;22:766–70. 10.1177/1078155215610915 26447099

[32] Patel JM Fox ER Zocchi M Lee ZE Mazer-Amirshahi M Trends in United States drug shortages for medications used in gastroenterology. Med Access Point Care. 2017;1:e58–64. 10.5301/maapoc.0000012

[33] Kweder SL Dill S Drug shortages: the cycle of quantity and quality. Clin Pharmacol Ther. 2013;93:245–51. 10.1038/clpt.2012.235 23340474

[34] European Commission. Future-proofing pharmaceutical legislation – Study on medicine shortages – Final report (revised). Brussels, Belgium: Publications Office of the European Union; 2021. Available: https://data.europa.eu/doi/10.2875/211485. Accessed: 23 February 2024.

[35] Yang C Wu L Cai W Zhu W Shen Q Li Z Current situation, determinants, and solutions to drug shortages in Shaanxi Province, China: a qualitative study. PLoS One. 2016;11:e0165183. 10.1371/journal.pone.0165183 27780218 PMC5079602

[36] Dill S Ahn J Drug shortages in developed countries—reasons, therapeutic consequences, and handling. Eur J Clin Pharmacol. 2014;70:1405–12. 10.1007/s00228-014-1747-1 25228250

[37] Health Canada. Drug shortages in Canada: Fiscal year 2022 to 2023 in review. 2023. Available: https://www.canada.ca/en/health-canada/services/drugs-health-products/drug-products/drug-shortages/2022-2023-review.html. Accessed: 23 February 2024.

[38] Izutsu KI Ando D Morita T Abe Y Yoshida H Generic Drug Shortage in Japan: GMP Noncompliance and Associated Quality Issues. J Pharm Sci. 2023;112:1763–71. 10.1016/j.xphs.2023.03.006 36965844

[39] Lee J Lee HS Shin H Krishnan V Alleviating drug shortages: The role of mandated reporting induced operational transparency. Manage Sci. 2021;67:2326–39. 10.1287/mnsc.2020.3857

[40] Lücker F Seifert RW Building up resilience in a pharmaceutical supply chain through inventory, dual sourcing and agility capacity. Omega. 2017;73:114–24. 10.1016/j.omega.2017.01.001

[41] Benhabib A Ioughlissen S Ratignier-Carbonneil C Maison P The French reporting system for drug shortages: description and trends from 2012 to 2018: an observational retrospective study. BMJ Open. 2020;10:e034033. 10.1136/bmjopen-2019-034033 32139487 PMC7059530

[42] Zovi A Inserra C Piacenza M Stefania V Drug shortages and drug unavailability: Analysis from an Italian hospital. European Journal of Hospital Pharmacy. 2020;27:A19.

[43] Yang YT Socal M Bennett CL Addressing the Drug-Shortage Crisis in Oncology. JAMA Oncol. 2024;10:155–6. 10.1001/jamaoncol.2023.5722 38175651

[44] Nagano H Shin J-h Kunisawa S Fushimi K Nagao M Imanaka Y Impact of the cefazolin shortage on the selection and cost of parenteral antibiotics during the supply disruption period in Japan: A controlled interrupted time series analysis. J Infect Public Health. 2023;16:467–73. 10.1016/j.jiph.2023.01.021 36738690

[45] National Medical Products Administration. [Notice on the pilot project for settling production bases of essential drug with small market demand]. 2012. Available: https://www.nmpa.gov.cn/xxgk/fgwj/gzwj/gzwjyp/20121107120001888.html. Accessed: 23 February 2024. Chinese.

[46] National Health and Medicine Administration. [Implementation opinions on reforming and improving the supply mechanism of drugs on shortage]. 2017. Available: https://www.gov.cn/xinwen/2017-06/29/content_5206573.htm#:~:text=%E4%B8%BA%E8%B4%AF%E5%BD%BB%E8%90%BD%E5%AE%9E%E5%85%A8%E5%9B%BD%E5%8D%AB,%E5%87%BA%E4%BB%A5%E4%B8%8B%E5%AE%9E%E6%96%BD%E6%84%8F%E8%A7%81%E3%80%82. Accessed: 23 February 2024. Chinese.

[47] General Office of the State Council. [Opinions on further improving the work of ensuring supply and stabilizing prices of drugs on shortage]. 2019. Available: https://www.gov.cn/zhengce/content/2019-10/11/content_5438499.htm. Accessed: 23 February 2024. Chinese.

[48] Yang C Cai W Li Z Page AT Fang Y The current status and effects of emergency drug shortages in China: Perceptions of emergency department physicians. PLoS One. 2018;13:e0205238. 10.1371/journal.pone.0205238 30300412 PMC6177176

[49] Sen-Crowe B McKenney M Elkbuli A Medication shortages during the COVID-19 pandemic: Saving more than COVID lives. Am J Emerg Med. 2021;45:557–9. 10.1016/j.ajem.2020.07.044 32763102 PMC7378009

[50] Verma AA Pai M Saha S Bean S Fralick M Gibson JL Managing drug shortages during a pandemic: tocilizumab and COVID-19. CMAJ. 2021;193:E771–6. 10.1503/cmaj.210531 33952621 PMC8177913

[51] The Economist Intelligence Unit. Addressing medicine shortages in Europe taking a concerted approach to drive action on economic, manufacturing and regulatory factors. London, England: Economist Intelligence Unit; 2017. Available: http://graphics.eiu.com/upload/topic-pages/medicine-shortages/Addressing-medicine-shortages-in-Europe-EIU.pdf. Accessed: 23 February 2024.

[52] Miljković N Batista A Polidori P Kohl S Horák P Results of EAHP’s 2019 Medicines Shortages Survey. European Journal of Hospital Pharmacy. 2020;27:202–8. 10.1136/ejhpharm-2020-002341 32471816 PMC7335625

[53] Francas D Mohr S Hoberg K On the drivers of drug shortages: empirical evidence from Germany. Int J Oper Prod Manage. 2023;43:1520–38. 10.1108/IJOPM-09-2022-0581

[54] Nurse-Findlay S Taylor MM Savage M Mello MB Saliyou S Lavayen M Shortages of benzathine penicillin for prevention of mother-to-child transmission of syphilis: An evaluation from multi-country surveys and stakeholder interviews. PLoS Med. 2017;14:e1002473. 10.1371/journal.pmed.1002473 29281619 PMC5744908

[55] Fox ER Unguru Y Oncology drug shortages in the USA — business as usual. Nat Rev Clin Oncol. 2020;17:128–9. 10.1038/s41571-019-0318-x 31831853

[56] Gourd E Cancer care critically affected by USA drug shortages. Lancet Oncol. 2023;24:727. 10.1016/S1470-2045(23)00289-9 37331355

[57] Shafiq N Pandey AK Malhotra S Holmes A Mendelson M Malpani R Shortage of essential antimicrobials: A major challenge to global health security. BMJ Glob Health. 2021;6:e006961. 10.1136/bmjgh-2021-006961 34728479 PMC8565534

[58] LI Y WU Z HU X Analysis of drug use in pediatric patients from 78 sample hospitals in 7 regions of China from 2013 to 2014. China Pharmacy. 2016;12:4058–61.

[59] Hu A Li Y Ma A Fu Y Sheng Y Zhao M Investigation and Analysis of the Reasons for Drug Shortage from 26 Medical Institutions in China. China Pharmacy. 2017;12:3754–3678.

[60] Ji X Wang Q Xu J Ding L Zeng Y Li M Investigation and Suggestion on the Situation of Pediatric Drug Shortage in Third-level Hospitals from Ji-angsu Province. China Pharmacy. 2017;12:4617–20.

