# Supplementary material for: Trends of drug shortages in China from 2018 to 2020: A retrospective observational study
Source: J Glob Health. 2024 Oct 11;14:04225. doi: 10.7189/jogh.14.04225 (PMC11466498; doi:10.7189/jogh.14.04225)
Supplement: Online Supplementary Document [file jogh-14-04225-s001.pdf]

Online Supplementary Document

Title: Trends of drug shortages from 2018 to 2020 in China: A retrospective observational study

Authors: Tao Huang, Lin Bai, Huangqianyu Li, Luwen Shi, Xiaodong Guan

Contents

Figure S1. Data collection and processing and calculation of the three outcome measures......2

Table S1. Top ten drug products that reported most frequently from 2018 to 2020. 3

Table S2. Trends on number, geographical coverage and duration of reported shortages by different subgroups from 2018 to 2020. 4

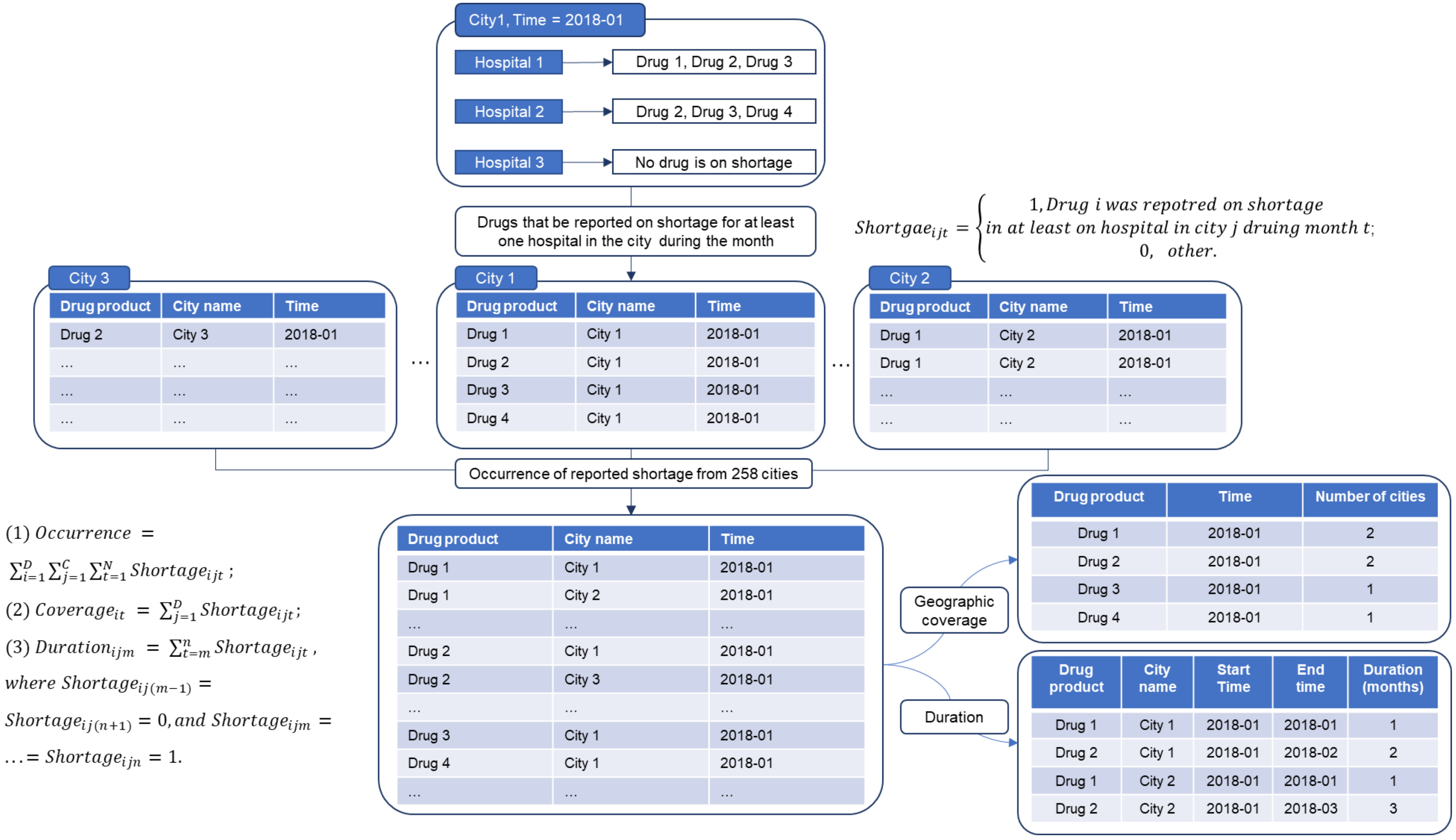

Figure S1. Data collection and processing and calculation of the three outcome measures.

Table S1. Top ten drug products that reported most frequently from 2018 to 2020

Generic name	Dosage form	Route of administration	Total occurrence of reported shortage
Methotrexate	Powder	Parenteral	1346
Allopurinol	Tablet	Oral	1280
Ketamine	Solutions	Parenteral	981
Cytarabine	Powder	Parenteral	951
Nitroglycerin	Tablet	Sublingual	892
Vincristine	Powder	Parenteral	698
Atropine	Solution	Parenteral	664
Vitamin K1	Solutions	Parenteral	633
Posterior Pituitary	Solution	Parenteral	612
Pyridostigmine Bromide	Tablet	Oral	599

Table S2. Trends on number, geographical coverage and duration of reported shortages by different subgroups from 2018 to 2020

Characteristics	Monthly number of shortages					Geographical coverage					Duration*				
	Z	*P* value	2018M1	2020M12	Change%	Z	*P* value	2018M1	2020M12	Change%	Z	*P* value	2018M7	2020M6	Change%
**All**	-3.83	<0.001	2655	2109	-20.6%	-2.02	0.043	2.5	2.5	0.3%	-3.10	0.002	2.9	2.6	-11.5%
**Emergency medicine**	0.90	0.367	238	281	18.1%	1.70	0.090	4.0	5.4	36.2%	-1.64	0.102	3.0	3.2	8.3%
**Dosage form**															
Aerosol	1.64	0.100	13	11	-15.4%	-0.15	0.881	1.4	1.2	-15.4%	0.43	0.670	1.3	2.3	72.5%
Capsule	-2.54	0.011	146	124	-15.1%	-2.59	0.010	1.7	1.9	9.8%	-1.36	0.172	2.6	2.5	-5.7%
Drop	-5.26	<0.001	5	1	-80.0%	-3.74	<0.001	1.0	1.0	0.0%	-1.65	0.099	1.0	1.0	0.0%
Emulsion	-1.22	0.223	28	35	25.0%	0.29	0.772	1.3	1.6	19.3%	-1.37	0.172	4.1	2.8	-33.6%
Gel	-0.62	0.537	8	20	150.0%	-2.69	0.007	1.0	1.2	17.6%	-0.67	0.503	1.3	2.1	60.7%
Granule	-3.05	0.002	64	26	-59.4%	-2.33	0.020	2.1	1.3	-39.1%	-0.62	0.535	1.5	2.2	50.5%
Liquid	1.17	0.243	3	6	100.0%	1.17	0.243	3.0	6.0	100.0%	-0.25	0.799	0.0	2.0	NA†
Miscellaneous	-0.92	0.357	0	3	NA†	-0.92	0.357	0.0	3.0	NA†	-2.50	0.012	2.0	0.0	-100.0%
Ointment	-2.84	0.004	45	31	-31.1%	-0.98	0.328	2.0	1.7	-12.0%	-2.43	0.015	3.6	2.9	-19.6%
Patch	0.36	0.716	1	2	100.0%	-1.17	0.241	1.0	1.0	0.0%	0.00	1.000	1.0	0.0	-100.0%
Pill	-3.39	0.001	3	2	-33.3%	-4.71	<0.001	1.5	1.0	-33.3%	-1.24	0.217	1.0	0.0	-100.0%
Powder	0.81	0.416	444	437	-1.6%	1.30	0.192	2.3	2.8	22.7%	-2.06	0.040	2.3	2.8	18.7%
Solution	-4.23	<0.001	1022	677	-33.8%	-5.79	<0.001	3.1	2.7	-11.0%	-1.46	0.143	2.8	2.7	-3.3%
Suppository	-1.36	0.173	14	9	-35.7%	0.34	0.733	1.0	1.5	50.0%	-0.42	0.673	1.8	3.8	117.1%
Suspension	-3.83	<0.001	21	9	-57.1%	-4.57	<0.001	2.3	1.5	-35.7%	-1.32	0.186	3.6	0.0	-100.0%
Syrup	0.26	0.796	3	3	0.0%	1.14	0.255	1.5	1.5	0.0%	-0.69	0.490	1.0	0.0	-100.0%
Tablet	-2.03	0.042	834	710	-14.9%	-2.24	0.025	2.5	2.6	5.1%	-3.40	0.001	3.3	2.3	-30.7%
Tincture	1.35	0.178	1	3	200.0%	0.97	0.331	1.0	1.5	50.0%	-1.95	0.052	1.0	0.0	-100.0%
**Route of administration**															
Buccal	0.51	0.609	1	3	200.0%	0.19	0.852	1.0	1.0	0.0%	-0.18	0.856	1.0	4.0	300.0%
Cutaneous	-1.48	0.139	76	94	23.7%	-2.22	0.026	1.7	1.6	-1.9%	-1.61	0.107	4.2	3.1	-25.9%
Hemodialysis	1.03	0.301	2	0	-100.0%	1.03	0.301	2.0	0.0	-100.0%	1.09	0.277	0.0	2.0	NA†

Characteristics	Monthly number of shortages					Geographical coverage					Duration*				
	Z	*P* value	2018M1	2020M12	Change%	Z	*P* value	2018M1	2020M12	Change%	Z	*P* value	2018M7	2020M6	Change%
Inhalation	-2.42	0.015	18	11	-38.9%	-2.12	0.034	1.5	1.2	-18.5%	0.10	0.921	1.5	1.7	11.1%
Nasal	1.04	0.300	5	8	60.0%	0.89	0.372	1.3	1.6	28.0%	0.96	0.336	1.3	2.4	82.1%
Ophthalmic	-3.64	<0.001	85	65	-23.5%	-8.04	<0.001	2.0	1.9	-6.1%	-2.11	0.035	2.4	1.9	-19.7%
Oral	-2.76	0.006	1092	885	-19.0%	-2.47	0.014	2.2	2.3	2.8%	-3.00	0.003	3.1	2.3	-24.3%
Otic	-0.77	0.444	4	2	-50.0%	-1.85	0.065	2.0	1.0	-50.0%	-2.28	0.023	1.0	0.0	-100.0%
Parenteral	-5.05	<0.001	1341	1007	-24.9%	-1.60	0.110	2.9	2.9	-0.2%	-2.11	0.035	2.7	2.7	1.7%
Rectal	-0.15	0.878	7	7	0.0%	-1.25	0.212	1.4	1.4	0.0%	-0.40	0.691	2.5	4.8	92.0%
Sublingual	-0.37	0.709	4	7	75.0%	-0.67	0.503	4.0	7.0	75.0%	-2.63	0.008	4.8	0.0	-100.0%
Vaginal	-1.54	0.123	20	20	0.0%	0.81	0.419	1.1	2.9	157.1%	-2.13	0.033	2.2	2.1	-4.0%
**Therapeutic area**															
Alimentary tract and metabolism	-2.76	0.006	375	289	-22.9%	-2.59	0.010	2.1	2.2	2.8%	-1.46	0.143	2.5	2.6	4.9%
Antiinfectives for systemic use	-3.22	0.001	346	243	-29.8%	-5.75	<0.001	2.5	2.1	-15.6%	-0.51	0.613	2.5	2.6	4.6%
Antineoplastic and immunomodulating agents	-0.13	0.900	256	236	-7.8%	-0.01	0.995	2.9	3.2	9.6%	-2.67	0.007	2.7	2.4	-11.9%
Antiparasitic products, insecticides and repellents	-2.48	0.013	15	9	-40.0%	-2.36	0.018	2.5	1.8	-28.0%	-0.27	0.784	2.4	4.0	65.5%
Blood and blood forming organs	-1.18	0.236	252	225	-10.7%	-0.10	0.054	2.9	3.3	13.9%	0.07	0.941	2.2	3.1	43.6%
Cardiovascular system	-0.74	0.458	311	268	-13.8%	-0.49	0.622	2.6	2.9	9.1%	-2.16	0.031	2.8	2.7	-3.9%
Dermatologicals	-1.28	0.201	88	89	1.1%	-3.72	<0.001	2.1	1.8	-15.4%	-1.79	0.074	4.3	3.1	-28.4%
Genito urinary	-2.23	0.026	110	74	-32.7%	-1.16	0.247	2.1	2.6	22.9%	-2.60	0.009	3.3	2.0	-40.8%

Characteristics	Monthly number of shortages					Geographical coverage					Duration*				
	Z	*P* value	2018M1	2020M12	Change%	Z	*P* value	2018M1	2020M12	Change%	Z	*P* value	2018M7	2020M6	Change%
system and sex hormones															
Musculo-skeletal system	-1.53	0.126	84	67	-20.2%	-1.02	0.306	1.9	2.2	13.2%	-3.47	0.001	4.9	2.0	-58.5%
Nervous system	-3.40	0.001	362	257	-29.0%	-4.33	<0.001	2.8	2.4	-13.6%	-2.80	0.005	3.0	2.3	-22.6%
Respiratory system	-4.12	<0.001	181	121	-33.1%	-6.65	<0.001	2.4	2.0	-14.2%	-0.05	0.960	3.6	2.6	-27.0%
Sensory organs	-3.15	0.002	97	75	-22.7%	-4.60	<0.001	1.9	1.8	-5.7%	-1.24	0.215	2.3	1.9	-18.9%
Systemic hormonal preparations, excl· sex hormones and insulins	-3.09	0.002	79	73	-7.6%	-1.50	0.135	2.7	3.0	11.7%	-2.36	0.018	3.6	2.5	-30.1%
Various	-0.54	0.593	99	83	-16.2%	2.36	0.018	2.5	3.5	36.2%	0.87	0.385	3.4	2.5	-25.1%

*To avoid possible bias stemming from the possibility that shortages starting earlier would expect longer duration, trend tests for duration of shortages were performed after excluding data from the first and last six months (almost one standard deviations of average shortage duration) of the study period.

†NA, not applicable when the denominator is zero.
